# Tetherin inhibits prototypic foamy virus release

**DOI:** 10.1186/1743-422X-8-198

**Published:** 2011-05-02

**Authors:** Fengwen Xu, Juan Tan, Ruikang Liu, Dan Xu, Yue Li, Yunqi Geng, Chen Liang, Wentao Qiao

**Affiliations:** 1 Key Laboratory of Molecular Microbiology and Biotechnology (Ministry of Education) and Key Laboratory of Microbial Functional Genomics (Tianjin), College of Life Sciences, Nankai University, Tianjin 300071, China; 2 Nebraska Center for Virology, University of Nebraska-Lincoln, NE 68583, USA; 3 McGill AIDS Centre, Lady Davis Institute- Jewish General Hospital, Montreal, Quebec, Canada H3T 1E2; Departments of Medicine and Microbiology and Immunology, McGill University, Montreal, Quebec, H3A 2B4, Canada

## Abstract

**Background:**

Tetherin (also known as BST-2, CD317, and HM1.24) is an interferon- induced protein that blocks the release of a variety of enveloped viruses, such as retroviruses, filoviruses and herpesviruses. However, the relationship between tetherin and foamy viruses has not been clearly demonstrated.

**Results:**

In this study, we found that tetherin of human, simian, bovine or canine origin inhibits the production of infectious prototypic foamy virus (PFV). The inhibition of PFV by human tetherin is counteracted by human immunodeficiency virus type 1 (HIV-1) Vpu. Furthermore, we generated human tetherin transmembrane domain deletion mutant (delTM), glycosyl phosphatidylinositol (GPI) anchor deletion mutant (delGPI), and dimerization and glycosylation deficient mutants. Compared with wild type tetherin, the delTM and delGPI mutants only moderately inhibited PFV production. In contrast, the dimerization and glycosylation deficient mutants inhibit PFV production as efficiently as the wild type tetherin.

**Conclusions:**

These results demonstrate that tetherin inhibits the release and infectivity of PFV, and this inhibition is antagonized by HIV-1 Vpu. Both the transmembrane domain and the GPI anchor of tetherin are important for the inhibition of PFV, whereas the dimerization and the glycosylation of tetherin are dispensable.

## Background

Retrovirus infection causes either acute or chronic diseases. To counteract, host cells have a variety of restriction factors to inhibit or block retrovirus infection. These include APOBECs (apolipoprotein B mRNA-editing catalytic polypeptides) [1,2], TRIM5α (tripartite motif protein 5-alpha) [3,4], TRIM28 (tripartite motif- containing 28) [5,6], ZAP (zinc-finger antiviral protein) [7,8] and tetherin [9,10].

Tetherin (also named BST-2, CD317, and HM1.24) has recently been described as a restriction factor in human cells, which can block the release of many enveloped viruses, such as retroviruses [9], filoviruses [11-13] and herpesviruses [14]. The expression of tetherin varies among different cell types and can be induced by type I interferon [9,10]. Some viruses have developed different strategies to overcome tetherin's restriction. Human immunodeficiency virus type 1 (HIV-1) Vpu and K5 of Kaposi's sarcoma-associated herpesvirus (KSHV) cause the degradation and cell surface downregulation of tetherin [9,14,15]. This can be achieved by targeting tetherin to the trans-Golgi network or to endosomes for proteasomal or lysosomal degradation by a β-TrCP-dependent mechanism [16-18]. HIV-2 Env and simian immunodeficieincy virus (SIV) Nef can reduce the expression of cell surface tetherin [19-21].

Tetherin is a 30-36 kDa type II transmembrane protein. It consists of four domains, an N-terminal cytoplasmic tail (CT), a single transmembrane domain (TM), an extracellular coiled-coil domain and a C-terminal glycosyl phosphatidylinositol (GPI) anchor [22-25]. Tetherin forms stable homodimers and is modified by asparagine- linked glycosylation [24]. Tetherin of human origin contains five cysteine residues. The three cysteine residues at positions 53, 63 and 91 in the extracellular domain are involved in disulfide bond-linked dimerization of tetherin [22,24].

Foamy viruses, also known as spumaretroviruses, are unconventional enveloped retroviruses that compose the only genus in the *Spumaretrovirinae *subfamily of *Retroviridae*. Foamy viruses replication has similarities to both hepadnaviruses and conventional retroviruses [26]. In this study, to explore the relationship between tetherin and foamy viruses, we detected the release and infectivity of prototypic foamy virus (PFV) after transfecting 293T cells with tetherin and PFV infectious clone. A series of deletions and mutations of tetherin were constructed to demonstrate the importance of the special structures of tetherin for its antiviral function. Furthermore, the cDNA of tetherins from bovine and canine origin were cloned to elucidate whether the inhibition of tetherin to PFV is species-specific.

## Materials and methods

### Plasmids

PFV full-length infectious clone pcPFV was kindly provided by Maxine L. Linial [27]. Flag-HuTHN and Flag-AgmTHN was subcloned from pcDNA3.1-HuTHN and pcDNA3.1-AgmTHN into pCMV-Tag2B (Stratagene) vector. The BovTHN cDNA and CanTHN cDNA were amplified by reverse transcription (RT)-PCR from RNA samples that were extracted from fetal bovine lung (FBL) cells and a fetal canine thymus cell line (Cf2Th), followed by insertion into pCMV-Tag2B vector. The Flag-HuTHN delCT (20-180 aa), Flag-HuTHN delTM (47-180 aa), Flag-HuTHN delGPI (1-161 aa), Flag-AgmTHN delCT (26-182 aa), Flag-AgmTHN delTM (50-182 aa) and Flag-AgmTHN delGPI (1-159 aa) were constructed by inserting individual PCR fragment into pCMV-Tag2B vector. The human tetherin mutant C53/63/91A was provided by Klaus Strebel. HuTHN C53/63/91A was subcloned into pCMV-Tag2B vector. HuTHN mutants with cysteine or asparagine to alanine substitutions, C53A, C63A, C91A, C53/63A, C63/91A, C53/91A, N65/92A and C3A/N2A, were generated from Flag-HuTHN and Flag-HuTHN C53/63/91A, using a QuikChange site-directed mutagenesis kit (Stratagene) (Table 1). All the new constructs were verified by sequencing.

**Table 1:** Descriptions of used plasmids

Name	Descriptions
Flag-HuTHN, Flag-AgmTHN, Flag-BovTHN, Flag-CanTHN	Human, African green monkey, bovine and canine tetherin expression plasmids
Flag-HuTHN delCT,	Human tetherin 20-180 aa expression plasmid
Flag-HuTHN delTM	Human tetherin 47-180 aa expression plasmid
Flag-HuTHN delGPI	Human tetherin 1-161 aa expression plasmid
Flag-AgmTHN delCT	African green monkey tetherin 26-182 aa expression plasmid
Flag-AgmTHN delTM	African green monkey tetherin 50-182 aa expression plasmid
Flag-AgmTHN delGPI	African green monkey tetherin 1-159 aa expression plasmid
HuTHN C53A, HuTHN C63A, HuTHN C91A	Human tetherin single cysteine mutated to alanine
HuTHN C53/91A, HuTHN C53/63A, HuTHN C63/91A	Human tetherin two of the three cysteines mutated to alanines
HuTHN C53/63/91A	Human tetherin all three cysteines mutated to alanines
HuTHN N2A	Human tetherin two asparagines at 65 and 92 mutated to alanines
HuTHN C3A/N2A	Human tetherin all three cysteines and two asparagines mutated to alanines

### Cells, viruses, and reagents

293T, BHK-21, and PFV indicator cell line (PFVL) cells were maintained in Dulbecco's modified Eagle's medium supplemented with 10% fetal calf serum, 50 IU/ml penicillin, and 50 μg/ml streptomycin at 37°C in humidified air with 5% CO2. The indicator cell line PFVL was created by transfecting BHK-21 with reporter plasmid containing the firefly luciferase gene driven by a PFV long terminal repeat promoter. At 48 h post-transfection, the medium was changed with the addition of 600 μg/ml neomycin (G418, Promega) for additional three weeks. The cells were replaced with fresh medium every three days. To obtain positive stable monoclones, G418-resistant cells were isolated further by plating them at a limiting dilution on to 96-well plates. 293T cells were transfected with pcPFV proviruses along with either wild type tetherin or mutants. Cell lysates and viral supernatants were collected at between 44 and 48 h posttransfection. Transient transfection was performed using 1 mg/ml polyethyleneimine (PEI) (Sigma) as previously described [28].

### Western blotting

Cells were washed twice with PBS, suspended in PBS, and mixed with an equal volume of sample buffer (4% sodium dodecyl sulfate, 125 mM Tris-HCl, pH 6.8, 10% 2-mercaptoethanol, 10% glycerol, and 0.002% bromophenol blue). For analysis of cysteine mutants under non-reducing conditions, cells were suspended in PBS and mixed with an equal volume of sample buffer that did not contain 2-mercaptoethanol. Proteins were solubilized by boiling for 20 min at 95°C. Western blot analyses were performed using anti-Gag polyclonal rabbit serum (provided by Maxine L. Linial) at a 1:5,000 dilution. The commercially available anti-Flag monoclonal antibody (Stratagene) was used at a 1:5,000 dilution. Anti-β-actin monoclonal mouse antibody (Sigma-Aldrich) was used at a 1:2,000 dilution as a loading control. After hybridizing the membrane with secondary goat anti-rabbit antibody and goat anti-mouse antibody at a 1:10,000 dilution, enhanced chemiluminescence reagents (Millipore) were used for signal detection with X-ray film.

For the preparation of viral proteins from supernatants of transfected cells, viral supernatants were collected, centrifuged at 2,000 rpm for 10 min to remove cell debris, and then pelleted through a 20% sucrose cushion for 2 h at 24,000 rpm and 4°C (Beckman). Viral pellets were resuspended in PBS and mixed with SDS sample buffer prior to loading onto a 10% protein gel. After proteins were separated by electrophoresis, they were transferred to nitrocellulose membranes (GE). The membranes were then blocked using Odyssey blocking buffer (LI-COR Biosciences) and incubated with anti-Gag polyclonal rabbit serum. Odyssey secondary antibodies were added according to the manufacturer's instructions (goat anti-rabbit IRDye 680). Blots were imaged using an Odyssey Infrared Imaging System (LI-COR Biosciences). Scan resolution of the instrument ranges from 21 to 339 μm, and in this study blots were imaged at 169 μm. Quantification was performed on single channels with the analysis software provided (LI-COR Biosciences).

### Luciferase assay

The indicator cells were plated in 96-well plates (10^4^/well) and cultured at 37°C for 18 h. Then cells were infected with viral supernatants. After replacing the virus stock with the maintenance medium, cells were incubated at 37°C for 48 h. The luciferase activity was measured by using a 96-channel chemiluminescense measurement machine Glomax (Promega, USA). Readout was counts per second. The relative light unit was determined automatically.

### Viral DNA copy number assay

The viral supernatants were collected at between 44 and 48 h posttransfection, centrifuged at 2,000 rpm for 10 min to remove cell debris, and pelleted through a 20% sucrose cushion for 2 h at 24,000 rpm and 4°C (Beckman). After resuspending viral pellets in PBS, viral DNA was extracted using QIAamp UltraSens Virus Kit (QIAGEN). Then viral DNA was quantified by real-time PCR using iQ SYBR green supermix (Bio-Rad). To generate a standard curve for cycle thresholds versus copy numbers, the pCMVTag-Gag plasmid was serially diluted to known concentrations. Primers for the amplification of *gag *gene were 5'-AATAGCGGGCGGGGACGACA- 3' and 5'-ATTGCCACGCACCCCAGAGC-3'. The DNA copy number was calculated using Bio-Rad iCycler software (version 3.1).

## Results

### Human tetherin inhibits the release of PFV

**Figure 1:**
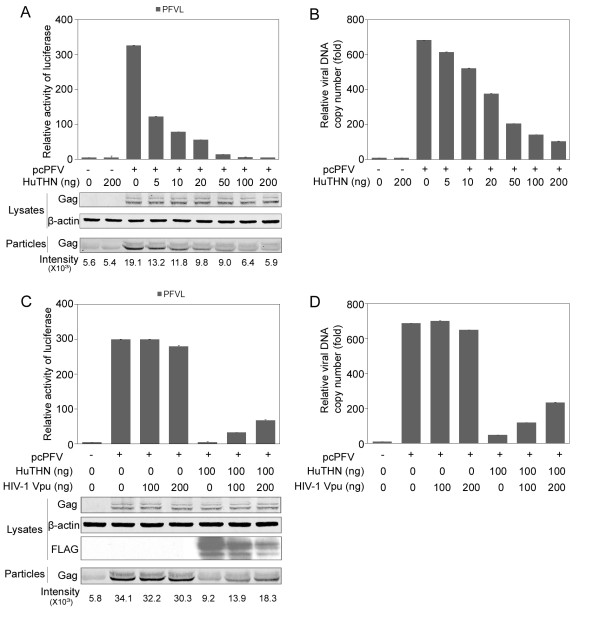
**Tetherin inhibits the release of PFV**. (A, B) 293T cells were transfected with PFV infectious clone pcPFV along with human tetherin expression plasmids (HuTHN). At 44-48 h after transfection, the amount of virus in supernatants was analyzed by infecting an indicator cell line PFVL, Western blotting (A), and viral DNA copy number assay (B). (C, D) 293T cells were transfected with pcPFV, HuTHN, and HIV-1 Vpu expression plasmid. The virus titer in the supernatants was analyzed by infecting PFVL. The luciferase activity and viral DNA copy number were normalized to the samples transfected with empty vectors. The cell lysates were immunoblotted with anti-Gag antibody. The same blots were reprobed with anti- β-actin antibody and anti-FLAG antibody. The viral particles were immunoblotted with anti-Gag antibody and Odyssey goat anti-rabbit IRDye 680. The intensity showed below the blots was performed with LI-COR analysis software.

We first examined whether human tetherin inhibits PFV release. To this end, we transfected 293T cells with the full-length PFV DNA construct named pcPFV and a human tetherin cDNA plasmid HuTHN. At 48 h after transfection, the amount of virus in supernatants was analyzed by Western blotting of Gag protein, viral DNA copy number assay and by infecting an indicator cell line named PFVL. PFV production and infectivity was reduced by human tetherin in a dose dependent manner, without affecting the expression of cell-associated Gag protein (Figure 1A, B).

HIV-1 Vpu counteracts tetherin by causing downregulation of cell surface tetherin [10,16,29,30]. To determine whether the antiviral activity of tetherin against PFV is antagonized by Vpu, HIV-1 Vpu was co-expressed with pcPFV and human tetherin. As expected, Vpu enhanced the release of PFV in the presence of tetherin (Figure 1C, D). Together, these data suggest that human tetherin inhibits the release of PFV and this inhibition is overcome by HIV-1 Vpu.

### Both the transmembrane domain and the GPI anchor of human tetherin are important for the inhibition of PFV

**Figure 2:**
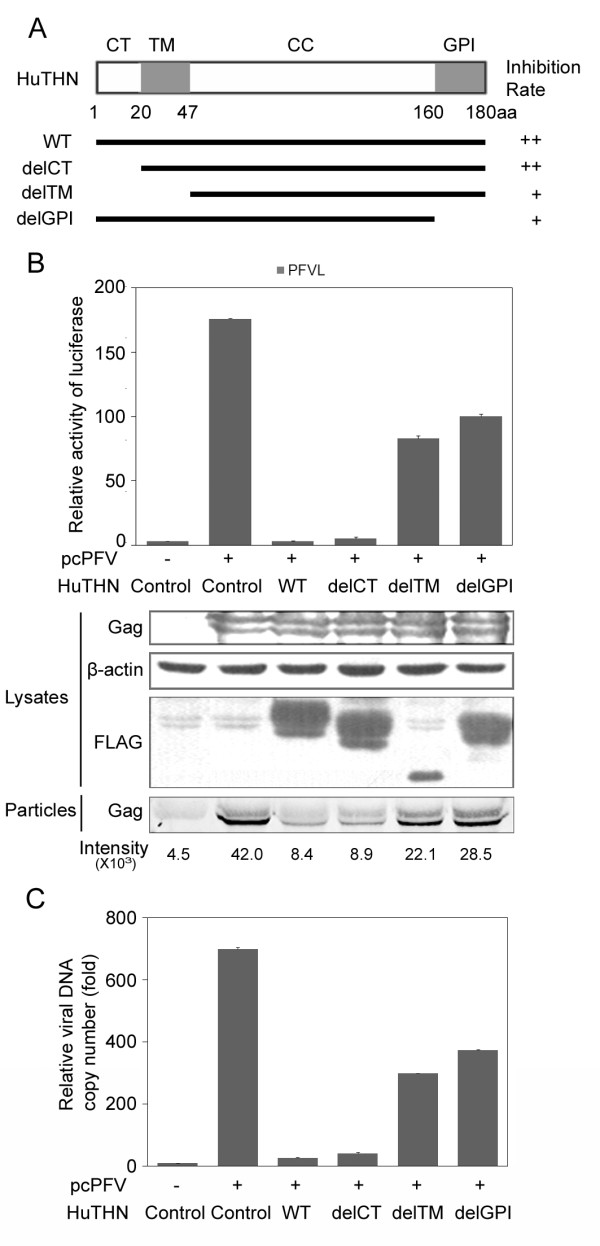
**TM domain and GPI domain of human tetherin are important for the inhibition of PFV**. (A) Schematic representations of human tethrin (HuTHN) and deletion mutants. WT, wild type; delCT, HuTHN (20-180 aa); delTM, HuTHN (47-180 aa); delGPI, HuTHN (1-161 aa). (B, C) pcPFV was transfected into 293T cells together with the indicated HuTHN wild type or truncated mutants. The amount of virus in supernatants was analyzed by infecting PFVL (B), Western blotting (B), and viral DNA copy number assay (C). The luciferase activity and viral DNA copy number were normalized to the samples transfected with empty vectors (control). The cell lysates were immunoblotted with anti-Gag antibody. The same blots were reprobed with anti- β-actin antibody and anti-FLAG antibody. The viral particles were immunoblotted with anti-Gag antibody and Odyssey goat anti-rabbit IRDye 680. The intensity showed below the blots was performed with LI-COR analysis software.

Tetherin was originally identified as a specific marker of late-stage B-cell maturation and a potential target for the immunotherapy of multiple myeloma [24,31]. It has an N-terminal transmembrane domain (TM) and a C-terminal GPI anchor. It associates with lipid rafts at the cell surface and on internal membranes [22]. In order to test whether these two domains are required for inhibition of PFV, we generated the cytoplasmic tail deletion mutant (delCT), transmembrane domain deletion mutant (delTM) and GPI deletion mutant (delGPI) (Figure 2A). The delCT mutant suppressed PFV production as efficiently as the wild type tetherin; in contrast, the delTM and the delGPI mutants only moderately inhibited PFV production (Figure 2B, C). These data suggest that the transmembrane domain and the GPI anchor of human tetherin are important for the inhibition of PFV.

### Dimerization and glycosylation of tetherin are not essential for inhibiting PFV

**Figure 3:**
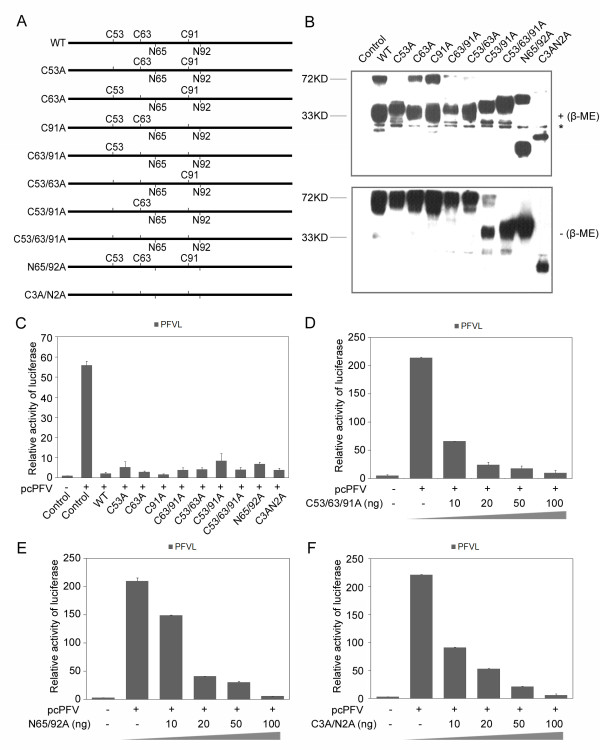
**The dimerization and glycosylation of human tetherin are not essential for its antiviral activity against PFV**. (A) Schematic representations of tetherin and various cysteine or asparagine to alanine mutants. (B) WT and mutant tetherin expressed in 293T cells were analyzed under both reducing and non-reducing conditions by Western blotting (*, non-specific band). (C) pcPFV was transfected into 293T cells together with the indicated human tetherin wild type or mutants. The titer of virus in supernatants was analyzed by infecting PFVL. (D-F) 293T cells were transfected with pcPFV and indicated 10, 20, 50, 100 ng of HuTHN mutants C53/63/91A (D), N65/92A (E), or C3A/N2A (F). The titer of virus in supernatants was analyzed by infecting the PFVL indicator cells.

Three cysteine residues C53, C63 and C91 contribute to tetherin dimerziation by forming disulfide bonds. To test their role in the antiviral activity of tetherin, we created three groups of mutants. The first group includes C53A, C63A and C91A that change each cysteine into an alanine. The second group includes C53/63A, C63/91A and C53/91A that change two cysteines into alanines. The third group is a C53/63/91A mutant that changes all three cysteines into alanines. We also mutated the two glycosylation sites at N65 and N92, and created the N65/92A mutant. Lastly, we generated the C3A/N2A mutant that changed the three cysteines and the two asparagines to alanines (Figure 3A). These mutants were expressed in 293T cells and the cell lysates were prepared in a buffer with or without β-ME. As shown in Figure 3B, all mutants showed a dominant 27-35 kDa monomer pattern under the reducing condition (+β-ME), while N65/92A and C3A/N2A reduced the apparent molecular weight to 17-20 kDa due to the lack of glycosylation. However, under the non-reducing condition, all mutants, except for C53/63/91A and C3A/N2A, formed the 60-70 kDa dimer. This result suggests that mutation of any individual cysteine or any two of the three cysteines in combination does not abolish tetherin dimerization. We next tested whether these mutants were able to inhibit PFV production. Since the mutants were expressed at different levels when the same amounts of plasmid DNA were transfected into 293T cells (Figure 3B), we thus adjusted the amount of plasmid DNA used for transfection such as to achieve similar expression levels of different mutants in order to compare the effect of these mutants on PFV release (data not shown). Interestingly, all mutants suppressed PFV production efficiently (Figure 3C). Furthermore, mutants C53/63/91A, N65/92A and C3A/N2A inhibited PFV release in a dose dependent manner (Figure 3D-F). These results indicate that dimerization and glycosylation are not essential for human tetherin to inhibit PFV release.

### Tetherin from simian, bovine and canine inhibit the release of PFV

**Figure 4:**
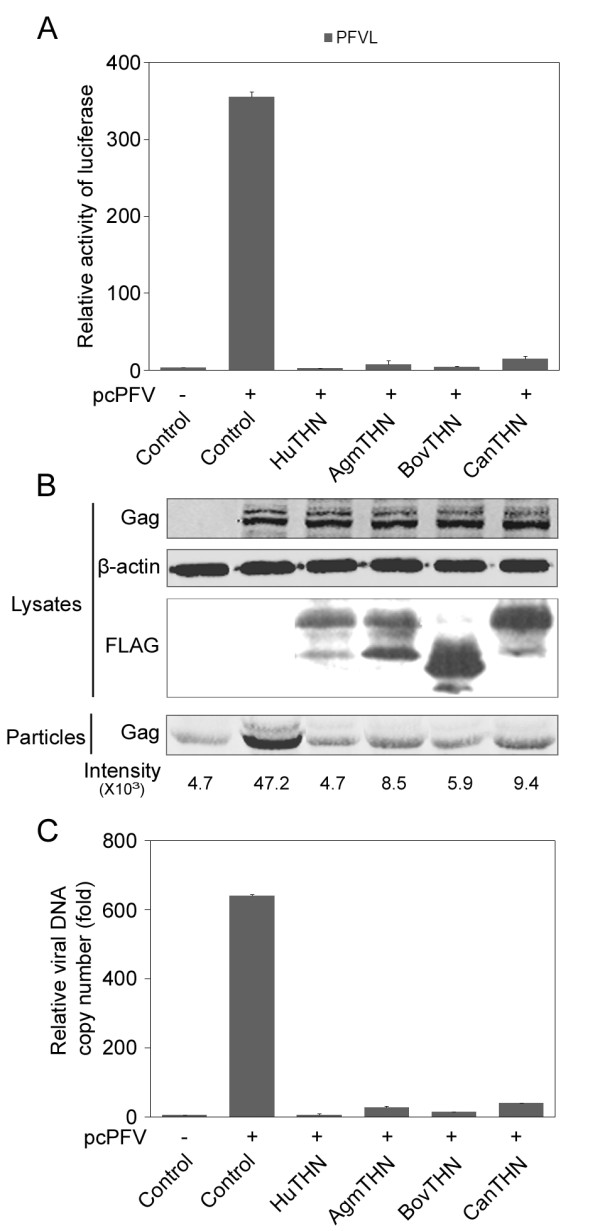
**Not only human tetherin, but also tetherins from simian, bovine and canine inhibit the release of PFV**. 293T cells were transfected with pcPFV- along with human tetherin (HuTHN), African green monkey tetherin (AgmTHN), bovine tetherin (BovTHN), or canine tetherin (CanTHN). At 44-48h after transfection, the amount of virus in cell lysates and supernatants was analyzed by infecting PFVL (A), Western blotting (B), and viral DNA copy number assay (C). The FLAG-tag tetherin and Gag proteins were immunoblotted with the antibodies indicated on the left side of each blot. The blot for β-actin was performed as an internal control. The luciferase activity and viral DNA copy number were normalized to the samples transfected with empty vectors (control).

**Figure 5:**
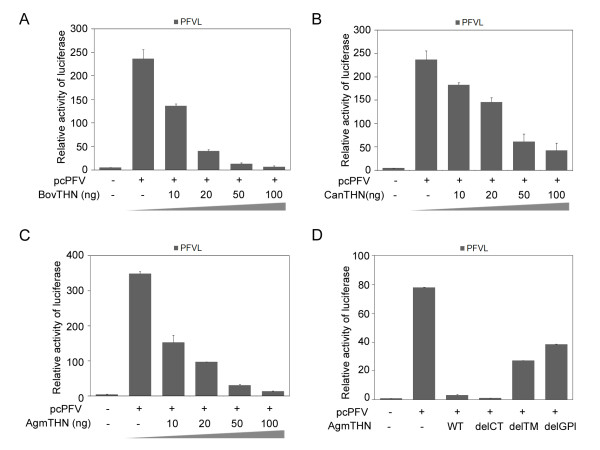
**Tetherins inhibit the release of PFV in a dose-dependent manner**. Both the TM domain and GPI anchor of AgmTHN are important for the inhibition to PFV. (A-C) pcPFV was transfected into 293T cells together with the indicated 10, 20, 50, 100 ng of BovTHN (A), CanTHN (B), or AgmTHN (C). The titer of virus in supernatants was analyzed by PFVL. (D) 293T cells were transfected with pcPFV, together with the indicated AgmTHN wild type or truncation mutants. WT, wild type AgmTHN; delCT, AgmTHN (26-182 aa); delTM, AgmTHN (50-182 aa); delGPI, AgmTHN (1-159 aa). The titer of virus in supernatants was analyzed by infecting PFVL.

PFV productively infects various cell lines that are derived from human, chimpanzees, monkeys, cows and dogs. We thus wished to test whether tetherins from these latter species could also inhibit PFV production. We cloned the cDNA of tetherins from bovine and canine from fetal bovine lung (FBL) cells and a fetal canine thymus cell line (Cf2Th). Tetherins of human, simian, bovine or canine origin displayed 59.5% identity at the amino acid level. To measure their ability to suppress PFV release, pcPFV was cotransfected with cDNA clones of human tetherin, African green monkey tetherin, bovine tetherin, or canine tetherin into 293T cells. The results show that all tetherins tested significantly inhibit the release of PFV (Figure 4) and that the inhibition effect of tetherin is dose-dependent (Figure 5A-C). Furthermore, both the TM domain and the GPI anchor of African green monkey tetherin are important for the inhibition of PFV (Figure 5D).

## Discussion

Foamy viruses are subject to restriction by several host factors. For example, APOBEC3G was reported to inhibit PFV, simian foamy virus (SFV) and feline foamy virus (FFV), and this restriction was counteracted by the Bet protein [32-34]. Trim5α could also restrict PFV, SFV, or FFV by targeting viral Gag protein [35]. In this study, we report that tetherin inhibits the release of PFV without affecting the expression of cell-associated Gag protein. Tetherin has been reported to block the release of retroviral, filoviral, arenaviral and paramyxoviral virus-like particles (VLPs) [11,36]. Here we demonstrate that, the production of infectious PFV is inhibited by tetherin. HIV-1 Vpu was found to enhance the detachment of infectious virions from the cell surface by downregulating the level of tetherin at the host cell surface and reducing its total cellular level [10,29]. This effect of Vpu is not limited to HIV-1 but was shown to rescue other viruses such as Rous sarcoma virus (RSV), Moloney murine leukemia virus (MuLV), and Xenotropic murine leukemia virus related virus (XMRV), as well as unrelated enveloped viruses such as Ebola virus [11,13,37]. Here our results show that, vpu enhances the release of PFV in the presence of human tetherin, and is consistent with previous reports [11].

Tetherin is a type II transmembrane protein that has both an N-terminal transmenbrane (TM) domain and a C-terminal glycosyl phosphatidylinositol (GPI) anchor. The TM domain and GPI anchor of tetherin were reported to be essential for its antiviral function against HIV-1 [9]. Consistent with this, the mutant delTM, which lacks the N-terminal transmembrane domain and is retained in the cell membrane only by its GPI anchor, did not block PFV release effectively. Similar results were shown for the delGPI mutant in which the GPI anchor signal was removed. These results suggest that N-terminal transmembrane domain and C-terminal GPI anchor at either end of the coiled-coil domain are important for the inhibition of PFV.

Tetherin can form stable cysteine-linked homodimers and can be modified by N-linked glycosylation. Formation of cysteine-linked tetherin dimers was shown to be a functional requirement for inhibition of HIV-1 release [38,39]. However, dimerization of tetherin was not essential for inhibiting Lassa and Marburg viruses [40]. Our results showed that neither the formation of cysteine-linked dimer nor the asparagines-linked glycosylation was required for inhibiting PFV. The mutants, C3A (all three cysteines were mutated) that does not form cysteine-linked dimer, and C3A/N2A (all three cysteines and two asparagines were replaced with alanines) that is unable to form homodimers and is not modified by glycosylation, still exhibit potent antiviral activity against PFV. These suggest that the dimerization and glycosylation of tetherin are not important for its antiviral activity against PFV.

Tetherins from different species have been shown to possess different antiviral functions and have different sensitivities to various viral countermeasures [17,18,41-43]. HIV-1 release is inhibited by tetherins from a wide selection of mammalian species, including Old World monkeys, such as rhesus macaques [20,41], African green monkeys [18,44], and Mustached monkeys [42], as well as mice and rats [17,41]. However, tetherin from a species of New World owl monkey (*Aotus lemurinus griseimembra*) is unable to restrict HIV-1 [43]. For PFV, it has a wide host range and can be isolated from saliva in infected animals. We thus cloned the bovine and canine tetherin and demonstrated that not only human tetherin, but also the simian, bovine and canine tetherin could inhibit the release of PFV.

Several models have been proposed to elucidate the possible mechanisms behind the antiviral activity of tetherin [9,39]. Some models indicated that tetherin might function as a direct physical tether bridging virions and the plasma membrane. Other models predicted that tetherin might tether virions and cells through some interactions with viral proteins or cellular proteins. However, a completely artificial tetherin-like protein using domains from other proteins, lacking significant sequence homology to native tetherin, could mimic the biological activity of native tetherin [39]. Also, immunoelectron microscopy performed on HIV-infected T cells demonstrated that tetherin formed an apparent physical link between virions and connected virions to the plasma membrane [45]. These data strongly suggest that tetherin directly tethers viral particles to cellular membranes, and that the tethering activity does not involve interactions with any specific cellular or viral cofactors, signaling or other indirect mechanisms. Consistent with these models, our results showed the major structural features of tetherin, the two membrane anchors were essential for the inhibition to PFV.

Tetherin may retain virus particles by two mechanisms. In one mechanism, one pair of membrane anchors of tetherin dimer are incorporated into virus, and the other pair still remain in cell membrane. In the other mechanism, the two membrane anchors of one monomer tetherin are embedded into virus. The anchors of the other monomer are in cell membrane. In this latter mechanism, the dimerization is necessary for the antiviral activity. In our research, the mutation which was unable to form homodimers still could inhibit the release of PFV effectively, the dimerization is thus not essential for the inhibition to PFV. This observation supports the first model.

Recently, it was demonstrated that tetherin/BST-2 not only inhibited release of Vpu-deleted HIV-1 particles by tethering them at the cell surface, but also impaired the infectivity of the progeny virions. The latter defect was a result of incomplete viral Gag processing and deficient maturation of HIV-1 particles caused by tetherin [46]. In our research, the infectivity of PFV from cells transfected with tetherin was also reduced significantly. Therefore, more studies will be necessary to elucidate how tetherin abrogates the infectivity of PFV.

## Conclusions

This study demonstrate that for foamy viruses, the special DNA viruses of retroviruses, tetherin of human, simian, bovine or canine origin inhibits the production of infectious PFV, and similar to HIV-1, the inhibition of human tetherin to PFV is counteracted by HIV-1 Vpu. We also find that both the transmembrane domain and the GPI anchor of tetherin, but not dimerization and glycosylation, are essential for the inhibition of PFV. These results suggest that the major structural features of tetherin, the two membrane anchors are essential for its antiviral activity. As the dimerization deficient mutants still exhibit potent inhibition effect on PFV production, our study supports a mechanism that one pair of membrane anchors of tetherin dimer are incorporated into virus, and the other pair remain in cell membrane to bridge the viruses and cells.

## Abbreviations

PFV: prototypic foamy virus; HIV: human immunodeficiency virus; GPI: glycosyl phosphatidylinositol; APOBECs: apolipoprotein B mRNA-editing catalytic polypeptides; TRIM5α: tripartite motif protein 5-alpha; TRIM28: tripartite motif- containing 28; ZAP: zinc-finger antiviral protein; KSHV: Kaposi's sarcoma- associated herpesvirus; SIV: simian immunodeficieincy virus; CT: cytoplasmic tail; TM: transmembrane domain; PFVL: PFV indicator cell line; FBL: fatal bovine lung cells; Cf2Th: fetal canine thymus cell line; SFV: simian foamy virus; FFV: feline foamy virus; VLPs: virus-like particles; RSV: Rous sarcoma virus; MuLV: Moloney murine leukemia virus; XMRV: Xenotropic murine leukemia virus related virus.

## Competing interests

The authors declare that they have no competing interests.

## Authors' contributions

FX and JT conceived and designed the study, performed data analysis and drafted the manuscript. DX contributed to plasmids construction and site-directed mutagenesis. RL and YL contributed to data analysis. CL, YG, and WQ analyzed the data and participated in manuscript redaction. WQ supervised the project, participated in the design of the study and data interpretation, and helped draft the manuscript. All authors read and approved the final manuscript.
